# Parasitics Impact on the Performance of Rectifier Circuits in Sensing RF Energy Harvesting

**DOI:** 10.3390/s19224939

**Published:** 2019-11-13

**Authors:** Antonio Alex-Amor, Javier Moreno-Núñez, José M. Fernández-González, Pablo Padilla, Jaime Esteban

**Affiliations:** 1 Departamento de Lenguajes y Ciencias de la Información, Universidad de Málaga, 29071 Málaga, Spain; 2 Information Processing and Telecommunications Center, Universidad Politécnica de Madrid, 28040 Madrid, Spain; jmn197@correo.ugr.es (J.M.F.-G.); jesteban@etc.upm.es (J.E.); 3 Departamento de Teoría de la Señal, Telemática y Comunicaciones, Universidad de Granada, 18071 Granada, Spain; jmfdez@gr.ssr.upm.es (J.M.-N.); pablopadilla@ugr.es (P.P.)

**Keywords:** RF energy harvesting, guidelines, sensor applications, low-power, Cockcroft–Walton multiplier, half-wave rectifier, parasitics modeling

## Abstract

This work presents some accurate guidelines for the design of rectifier circuits in radiofrequency (RF) energy harvesting. New light is shed on the design process, paying special attention to the nonlinearity of the circuits and the modeling of the parasitic elements. Two different configurations are tested: a Cockcroft–Walton multiplier and a half-wave rectifier. Several combinations of diodes, capacitors, inductors and loads were studied. Furthermore, the parasitics that are part of the circuits were modeled. Thus, the most harmful parasitics were identified and studied in depth in order to improve the conversion efficiency and enhance the performance of self-sustaining sensing systems. The experimental results show that the parasitics associated with the diode package and the via holes in the PCB (Printed Circuit Board) can leave the circuits inoperative. As an example, the rectifier efficiency is below 5% without considering the influence of the parasitics. On the other hand, it increases to over 30% in both circuits after considering them, twice the value of typical passive rectifiers.

## 1. Introduction

With the rapid development of wireless communications in the latest years, there has been an exponential increase in the number of radiofrequency (RF) transmitters operating in VHF and UHF bands. As a consequence, ambient RF power density has increased and RF energy harvesting has become an environment-friendly energy source for low-consumption electronic devices, such as sensors, regulators, oscillators and LCD screens [1,2]. Additionally, RF energy is present both indoors and outdoors, which is an advantage with respect to other harvestable sources. The most significant power contribution normally comes from mobile network (0.8, 0.9, 1.8, and 2.1 GHz) and WiFi frequency bands (2.4 and 5.2 GHz). Specifically, previous works have demonstrated that most part of the incoming RF power has its origin in mobile bands [3,4,5]. Concretely, Ref. [5] points out that more than an 80% of the power harvested is located in 0.8 and 0.9 GHz frequency bands. 

**Figure 1:**
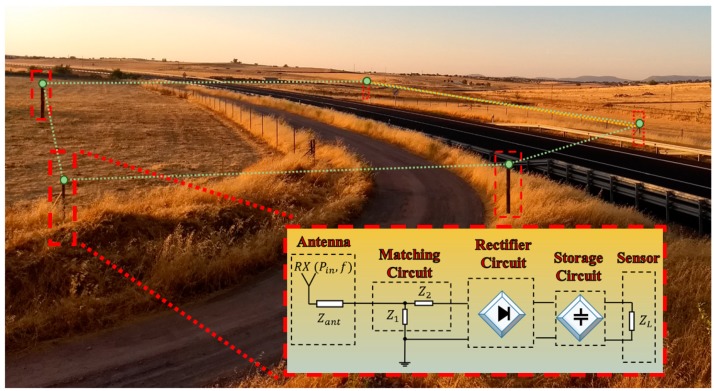
Example of a wireless sensor network (WSN) where the sensing devices are powered by radiofrequency (RF) energy harvesting systems.

Rural and remote areas are especially interested for looking at self-sustaining sensing systems due to the difficulty of access to them. Low-power sensors can benefit from the use of RF harvesting systems and be deployed in these challenging environments, forming wireless sensor networks (WSNs) [6] applied in, e.g., tracking and farming tasks [7,8] or monitoring fire risk areas [9]. As an example, Figure 1 shows a possible implementation of a low-power WSN formed by independent. 

Autonomous nodes. Each node is powered by a RF harvesting system, formed in four stages [10]: a RF harvester, a receiving antenna that collects energy from the bands of interest; a rectifier circuit that converts the signal acquired by the antenna into a DC supply source; a matching circuit, in charge of maximizing the power transfer from the antenna to the rectifier circuit; and a storage circuit, which stores the acquired power in order to feed a certain electronic device.

Nowadays, the bottleneck in energy harvesting is the design of the rectifier circuit. This is partially attributed to the losses present in the diodes. The low input power level provokes the diodes into dissipating an important part of the incoming power. Specifically, RF harvesting systems must operate with lower power densities [3,4,5] compared to other harvestable sources, such as the sun, wind or vibrations [11]. Moreover, the nonlinearity associated with the diodes represents an added difficulty [12]: the performance of the entire system is dependent, among other parameters, on the input power, which unfortunately hampers the design of the matching circuit. Because of these combined facts, the efficiency of different topologies of passive circuitries (not externally fed) is normally under 15% [10,12,13,14]. On the other hand, there are some recent studies that have applied active circuits (externally fed) [14,15] in order to lower the threshold voltage of diodes/transistors. In these cases, the component losses are reduced and the efficiency enhanced, at the expense of using an external feed, which is prohibitive for self-sustaining devices [16,17].

The most commonly used topology for a passive rectifier circuit is the half-wave rectifier. It benefits from a simpler design and lower losses compared to full-wave schemes [18], since only one diode is required. On the other hand, the low-voltage input levels make multiplier circuits, such as the Cockcroft–Walton (CW) multiplier [14], an interesting option to consider. At least two diodes are needed in this configuration, so the losses are similar to those of a full-wave rectifier but higher compared to a half-wave rectifier. As opposed to the full-wave rectifier, the CW multiplier is capable of elevating the input voltage while rectifying. Ideally, the more stages are placed, the higher the output voltage is. However, the losses in the diodes typically prevent one from using more than one stage in the CW multiplier, since the efficiency rapidly decays.

In addition, there is a need for a circuit model that is capable of predicting how the parasitics affect the operation of the system. Regretfully, the vast majority of papers in the literature do not provide a clear insight on the matter. In this paper, we present a design methodology to maximize the power supplied to the sensor by taking into account the nonlinearity of the circuits and the effect of the parasitic elements. We study two different configurations: the half-wave rectifier and the CW multiplier. However, the steps followed in the document can be extrapolated, without loss of generality, to any other configuration. According to the estimations of the incoming power provided in [3,4,5], the circuits are designed to operate within 800–900 frequency bands, the center frequency being 870 MHz. Several combinations of components were tested in both circuits. Thus, the most harmful parasitics in the circuits were identified and modeled. The experimental results show that a proper modeling of the parasitic elements is fundamental in order to avoid a low conversion efficiency in the rectifiers.

The document is organized as follows. Section 2 presents the design methodology and the different steps involved in it. Section 3 gives useful insights on the choice of components. Section 4 describes the design of the matching circuit and the search for the optimal source impedance that maximizes the rectifier efficiency. Section 5 presents the model of the parasitics and their influence on the overall performance of the circuits. Finally, Section 6 presents the main conclusions.

## 2. Design Process

Due to the nonlinearity of rectifier circuits, their input impedance (and their response) is dependent on many parameters: the input power, the antenna impedance, the load impedance (the type of sensor we use), the operation frequency, etc. Furthermore, the rectifier cannot be directly attached to the antenna, since there exists an impedance mismatch between both, which translates into a low rectifier efficiency. Consequently, a matching circuit is commonly placed between the antenna and the rectifier to maximize the power transfer. However, the design of the matching circuit suffers from the same problems associated with the nonlinearity of the rectifier.

**Figure 2:**
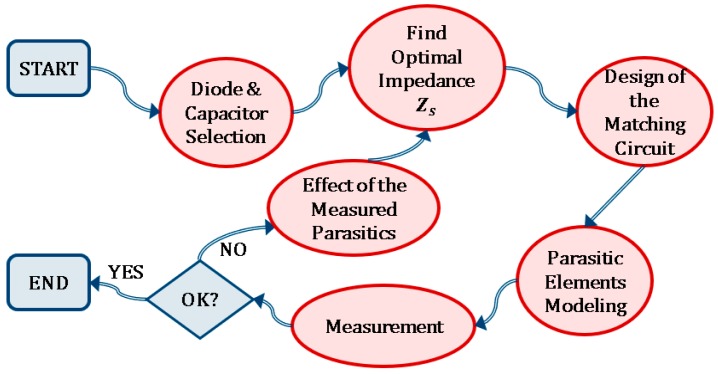
Flowchart for the efficient design of rectifier circuits.

The design method that overcomes these difficulties and maximizes the rectifier efficiency is presented in Figure 2, as an iterative process. First, we must select the components that are part of the rectifier circuit, trying to reduce the losses in the diode and the output voltage ripple of the rectified DC signal. Then, we must design the matching circuit. The design of the matching circuit is based on the search, for a given load $Z_{L},$ of the optimal source impedance $Z_{s}$ that maximizes the power transfer between the antenna and the rectifier. Thus, the components of the matching circuit are estimated in order to maximize the rectifier efficiency. However, the parasitics of the components and PCB (Printed Circuit Board) do influence the search of $Z_{s}.$ They should be taken into account in the circuits in order to avoid the frequency displacement they cause. Once they have been properly modeled, the circuit is measured in the laboratory. If the measurement does not correspond with the simulations, the optimal source impedance is recalculated, by taking now into account the effect of the parasitics that was measured, and the matching circuit is improved until acceptable results are achieved. 

The approach proposed here is not limited to any particular power or frequency range. Furthermore, complementary tools can be utilized in any particular stage of the design method. As an example, the vector network analyzer (VNA) can be utilized for hand-tuning the matching circuit. Trimmable (variable) components can be added to the matching circuit, so the efficiency of the rectifiers can be optimized with the VNA. However, this approach should be seen as an ad hoc solution, specific and unique for each design. As a consequence, it could be difficult to extract conclusions about the deviations that the parasitics cause and to quantify their effect on the circuit, which is the main purpose of the method we propose. Gaining insight on the parasitics’ impact could potentially ease the design of future efficient rectifiers. 

## 3. Choice of Components

In this section, some advice is given in order to select the components that are part of the rectifier circuit. The diode is the critical element in the circuit design. It must switch quickly to ensure its operation in the GHz range, and it must consume very little power. Compared to p–n diodes, Schottky diodes (metal–semiconductor (M–S) junctions) benefit from a faster switching time and a lower forward voltage drop (0.2−0.3 V versus 0.6−0.7 V), which makes them perfect for use in RF energy harvesting. Two diodes frequently used in this context are the HSMS-2822, specifically designed for input power levels above −20 dBm at frequencies below 4 GHz; and the HSMS-2850 (single mode), optimized for use with small signals ($P_{in}<-20$ dBm) at frequencies below 1.5 GHz. According to their datasheets [19], HSMS-2822 diodes can provide 0.1 mA with a maximum voltage drop of 0.22 V, while HSMS-2850 diodes can provide the same current with a maximum voltage drop of 0.15 V. Some studies point out that the power levels foreseen in RF energy harvesting are normally higher than −20 dBm [4,10,12,14]. Thus, we decided to use the HSMS 2822 diode.

The choice of the capacitor is a trade-off between the output ripple of the circuit, and the self-resonant frequency (SRF) of the capacitor itself. The higher the value of the capacitor, the lower the output ripple is, and normally, the lower its SRF. The SRF is directly related to the parasitics of a certain component and is calculated as

(1) $$\mathrm{SRF}\;\left[\mathrm{Hz}\right]=\frac{1}{2\pi \surd LC}$$

The lower the SRF is, the higher the value of the parasitic element and the more harmful its effect on the circuit. With the use of Equation (1), we show an estimate in Section 5 for the parasitic elements associated with inductors and capacitors.

## 4. Circuit Design

In this section, we describe the development of the procedure to design the lossless matching circuit that maximizes the rectifier efficiency. The design is particularized for the two topologies of the rectifiers under study: the CW multiplier and the half-wave rectifier. The matching circuit is implemented with a *L* network in both circuits. However, the same procedure is still applicable for different topologies of rectifier and matching schemes.

**Figure 3:**
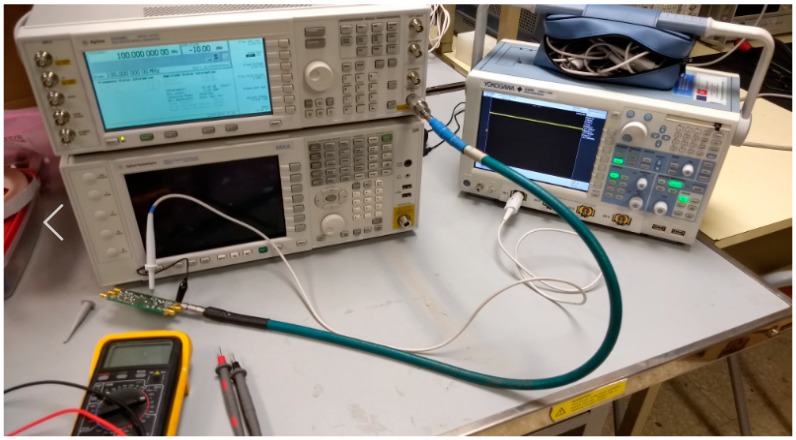
Measurement setup.

**Figure 4:**
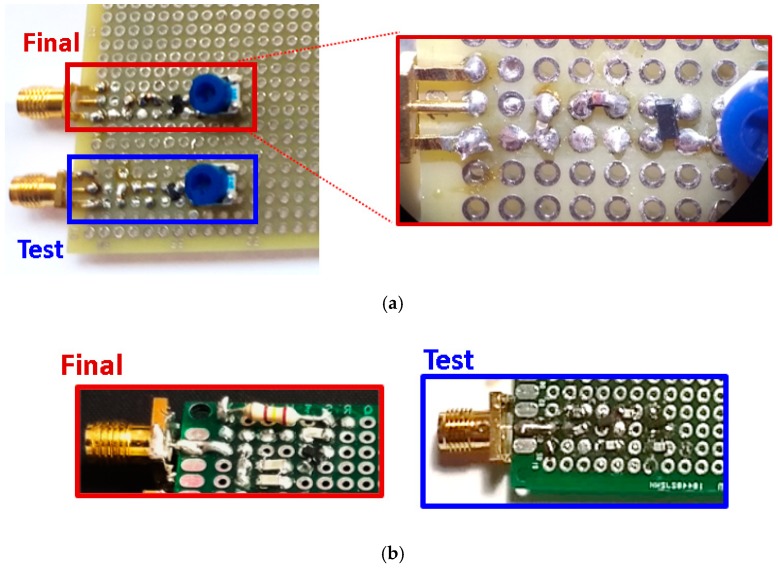
Manufactured (**a**) half-wave rectifiers and (**b**) Cockcroft–Walton multipliers. The final half-wave rectifier was observed under the microscope. Label “Test” refers to the test CW multiplier and half-wave rectifier used to study the effect of the parasitic elements, and label “Final” refers to the redesigned final CW multiplier and half-wave rectifier.

The measurement setup and the fabricated circuits are visualized in Figure 3 and Figure 4. The circuits were implemented in perforated boards: the components were placed in the top layer and the tracks in the bottom layer. Two different boards of similar characteristics were used in the design of the half-wave rectifiers and the Cockcroft–Walton multipliers. In Figure 4, label “Test” refers to the test CW multiplier and half-wave rectifier used to study the effect of the parasitic elements, and label “Final” refers to the redesigned final CW multiplier and half-wave rectifier.

### Optimal Source Impedance

**Figure 5:**
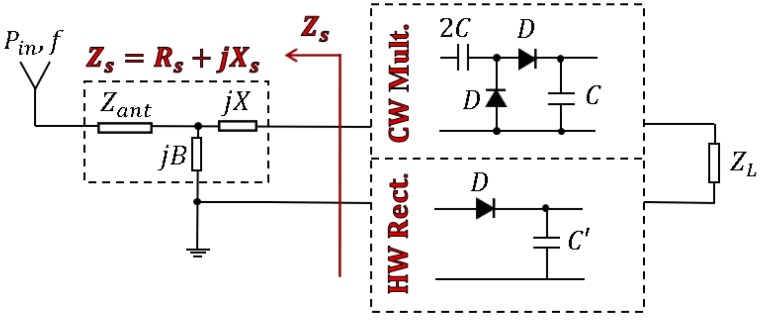
Search of the optimal source impedance $Z_{s}$ in the Cockcroft–Walton multiplier and the half-wave rectifier.

Usually, the input power of the circuit (the power acquired by the antenna) is a combination of carriers with different amplitudes and frequencies [4]. As illustrated in Figure 5, a smart approach can be applied in this scenario. The antenna impedance and the matching circuit were replaced together, by a source impedance $Z_{s}=R_{s}+jX_{s}.$ Afterwards, an *L* network was designed in order to transform the antenna impedance $Z_{ant}$ into the optimal source impedance $Z_{s}$ at the frequency of interest (870 MHz, in this case).

**Figure 6:**
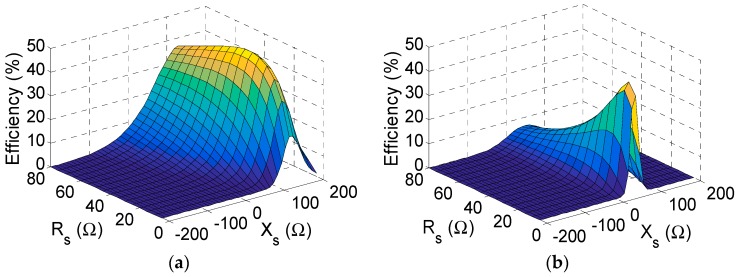
Efficiency with respect to the source impedance $Z_{s}=R_{s}+jX_{s}$ in the Cockcroft–Walton (CW) multiplier before (**a**) and after (**b**) considering the parasitics.

**Figure 7:**
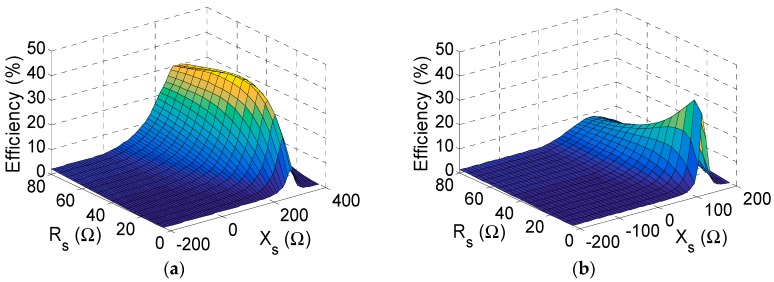
Efficiency with respect to the source impedance $Z_{S}=R_{s}+jX_{s}$ in the half-wave rectifier before (**a**) and after (**b**) considering the parasitics.

To make a fair comparison between the performances of both circuits (CW and half–wave rectifiers), the input powers and the frequencies for both were kept the same ($P_{in}=0$ dBm, f=870 MHz). However, the load was different, $Z_{L}=2.34$ kΩ in the CW and $Z_{L}=8$ kΩ in the half-wave rectifier. For the two given loads $Z_{L},$ the optimal source impedance $Z_{s}$ was found via an optimization process in a harmonic balance simulation [20] performed in commercial software *ADS*. Since $Z_{s}$ is a complex value, the efficiency of the rectifier may be visualized in a 3D plot with respect to the resistance $R_{s}$ and the reactance $X_{s}.$ Thus, Figure 6 and Figure 7 illustrate the search of the optimal $Z_{s}$ in.

The CW and in the half-wave rectifiers, respectively, before and after considering the parasitics.

Despite the optimal values being different ($Z_{s}=42+j120$ Ω and $Z_{s}=10+j50$ Ω in the CW; $Z_{s}=30+j280$ Ω and $Z_{s}=10+j130$ Ω in the half-wave rectifier), both figures share some similarities. First, the reactance $X_{s}$ is positive in all the cases. This points out that the rectifiers show a mainly capacitive behavior (negative reactance), which should be neutralized with a positive reactance in the matching circuit to enhance a rectifier’s efficiency. In addition, the optimal reactance $X_{s}$ decreases (but remains positive) after including the effect of the parasitics. This is due to the parasitic series-inductances associated with the via holes (see Section 5), which contribute with a positive reactance term and reduce the capacitive behavior of the rectifiers. Second, the shape of the 3D plot is completely different before and after considering the parasitic elements, which illustrates the importance of modeling them. Concretely, the curves show a steeper response after including the parasitics. This fact is directly related to the reduction of $R_{s}$ and indirectly indicates to us that the parasitic elements reduce the operation bandwidth of the circuit.

With the use of the formulas presented in [21], the optimal source impedance $Z_{s}$ is transformed into the lumped elements that form the *L* matching network. The antenna impedance $Z_{ant}$ was assumed to be $Z_{ant}=50$ Ω here. With the components available in the laboratory (Table 1), we implemented the L networks that can be seen in Section 5.2. The *L* network of the CW multiplier lacks a capacitor, as far as the optimal impedance $Z_{S}=42+j120\;\Omega$ can be approximated by $Z_{S}\approx 50+j120\;\Omega .$ Since the impedance of the antenna already covers 50 Ω, only a series inductance is needed (two in series, in our case, due to inventory shortage) to achieve j120 Ω at 870 MHz.

## 5. Parasitic Elements

In this section, we describe the parasitics that are associated with the lumped elements and the PCB. Thus, a circuit model was derived to correct the frequency displacement they cause. Furthermore, the contribution of each parasitic was quantified and the most harmful parasitics were identified.

### 5.1. Circuit Model

**Figure 8:**
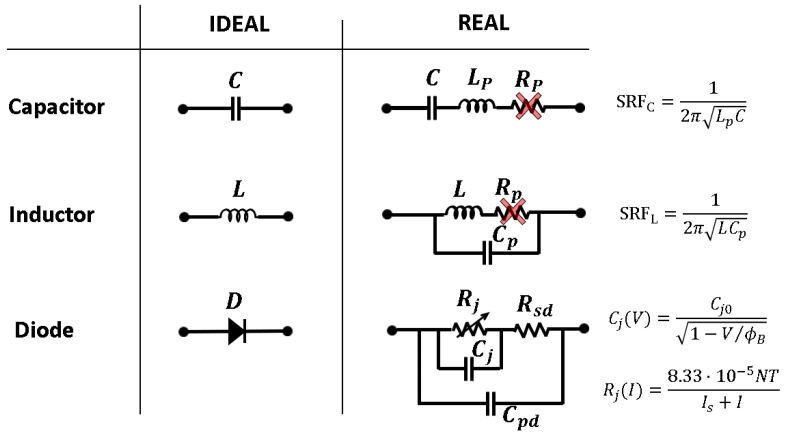
Model of the parasitics for the lumped elements.

**Figure 9:**
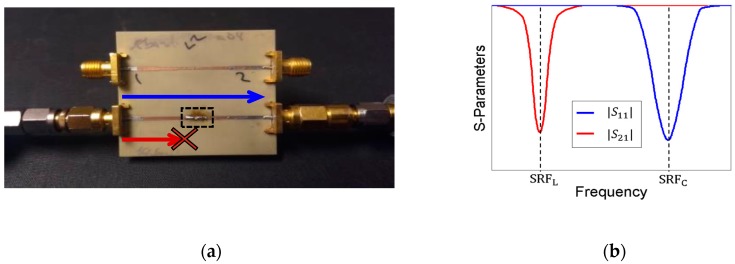
Extraction of the parasitic elements from inductors and capacitors through their self-resonant frequencies (SRFs): (**a**) measurement board and (**b**) S-parameters. The SRF of an inductor is determined via the non-transmission peak in the $\left|S_{21}\right|.$ The SRF of a capacitor is determined via the non-reflection peak in the $\left|S_{11}\right|.$

From previous studies [14] and our observations, the necessity of a robust model for the complete circuit is clear. Firstly, the SRF of the inductors and capacitors was estimated in the laboratory by connecting the component in series in a 50 Ω line. As described in Figure 8, capacitors and inductors are modeled as series and a shunt LC tank, respectively, since the effect of the parasitic resistances $R_{p}$ can be neglected. Their parasitics are related with the self-resonant frequency (SRF), according to Equation (1). Furthermore, their SRF can be measured in the laboratory with the 50 Ωline shown in Figure 9a and with the use of a network analyzer. In the case of the inductor, its SRF was determined via the non-transmission peak in the $\left|S_{21}\right|$ parameter (red curve in Figure 9b). In the case of the capacitor, its SRF was determined via the non-reflection peak in the $\left|S_{11}\right|$ parameter (blue curve in Figure 9b). Subsequently, the parasitic element of the component was obtained by using Equation (1). To avoid considering unwanted terms in the calculus (parasitics associated with the cables in the measurement, to the microstrip line, etc.), the system should be calibrated beforehand. Table 1 shows the parasitic elements of the lumped elements used in the CW multiplier and the half-wave rectifier.

The diode is also the critical element from the point of view of the parasitics. Ohmic losses in the diode are modeled through the series resistance $R_{sd}=7.8\;\Omega$ and the junction resistance $R_{j}$. The series resistance has little effect on the circuits, so it was neglected in this work. However, the junction resistance is dependent on the current I flowing through the diode: the lower the current is, the higher $R_{j}$. According to the datasheet of the diode, $R_{j}$ can be calculated as [19]:

(2) $$R_{j}\left(I\right)=\frac{8.33\cdot 10^{-5}NT}{I_{s}+I}\approx \frac{0.026}{I_{s}+I}\;@\;25\;{}^{{}^{\circ}}C$$

where $I_{s}$ is the saturation current, N is an ideality factor and T is the temperature. For the HSMS-2822 diode [19], $I_{s}=48$ nA, and N=1.067. For the current levels foreseen in the work (0.1–1 mA), we may expect junction resistances within the interval $R_{j}=26–260\;\Omega .$ The junction capacitance of a Schottky diode can be modeled by:

(3) $$C_{j}\left(V\right)=\frac{C_{j0}}{\sqrt{1-\frac{V}{\phi_{B}}}},$$

where $C_{j0}$ is the zero-bias junction capacitance and $\phi_{B}$ is the built-in potential. For the HSMS-2822 diode [19], $C_{j0}=0.65$ pF and $\phi_{B}=26.7$ V. For the voltage values foreseen in the article (V<2 V), we may expect junction capacitances within the interval $C_{j}=0.65-0.68$ pF. Additionally, the capacitive coupling between the metallic pins of the diode package can significantly affect the performance of the circuit. This term is modeled with a parallel parasitic capacitance $C_{pd},$ whose value is slightly tuned in simulation to $C_{pd}=0.75$ pF to fit the measurements.

The parasitics associated with the lumped components have already been considered and modeled. The main contribution from the PCB comes from the via holes. The parasitic inductance associated with the via holes can be estimated as in [22]:

(4) $$L\;\left[\mathrm{nH}\right]=5.08h\;\left(\ln\left(\frac{4h}{d}\right)+1\right),$$

where h is the height of the PCB and d the diameter of the hole, both in inches. Variations in the height of the PCB can modify the value of the parasitic inductance associated with the vias. On the other hand, variations in the diameter of the via have less influence on the parasitic inductance due to the logarithm involved in Equation (4). In the case of the Cockcroft–Walton multiplier, the height of the PCB is 1.61 mm, and the diameter is 1 mm, which leads to a theoretical parasitic inductance $L_{vTheory}=0.92$ nH. In the case of the half–wave rectifier, both the height of the PCB (1.51 mm) and the diameter of the hole (0.90 mm) are smaller, which leads to a smaller inductance $L_{vTheory}^{\prime }=0.88$ nH. In manufactured circuits, these values will be higher since the vias are not perfect cylinders. The effective height of the PCB is normally higher, and the welding process causes additional series inductances. Thus, the parasitic inductances associated with the via holes are slightly tuned in simulation, resulting in $L_{v}=1.30$ nH for the CW multiplier and $L_{v}^{\prime }=1.20$ nH for the half–wave rectifier. 

### 5.2. Discussion

**Figure 10:**
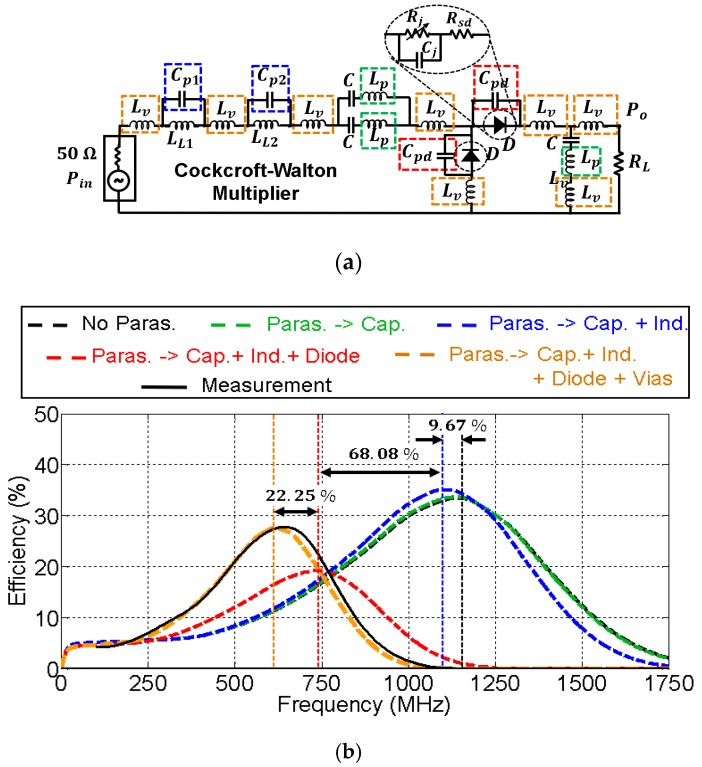
Model for the parasitic elements in the test Cockcroft–Walton multiplier (**a**) and their relevance in the efficiency of the circuit (**b**). The values of the components are: $L_{v}=1.30\;\mathrm{nH},$ $C_{p1}=0.10\;\mathrm{pF},$ $L_{L1}=4.3\;\mathrm{nH},$ $C_{p2}=0.19\;\mathrm{pF},$ $L_{L2}=8.2\;\mathrm{nH},$ C=33 pF, $L_{p}=0.16\;\mathrm{nH},$ $C_{pd}=0.75\;\mathrm{pF},$ and $R_{L}=2.34\;k\Omega .$ The input power was $P_{in}=0$ dBm. Black and green dashed lines overlap.

**Figure 11:**
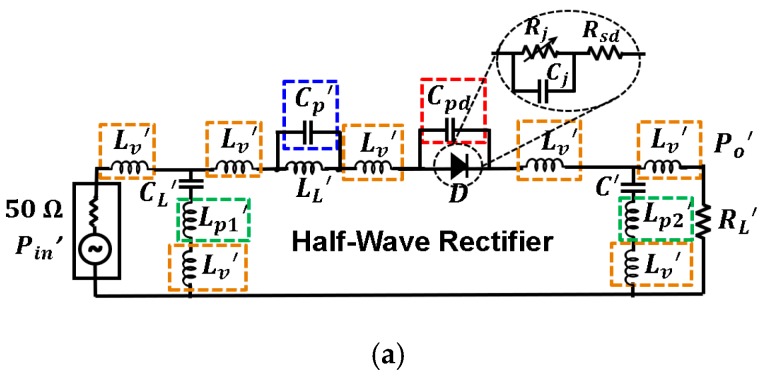

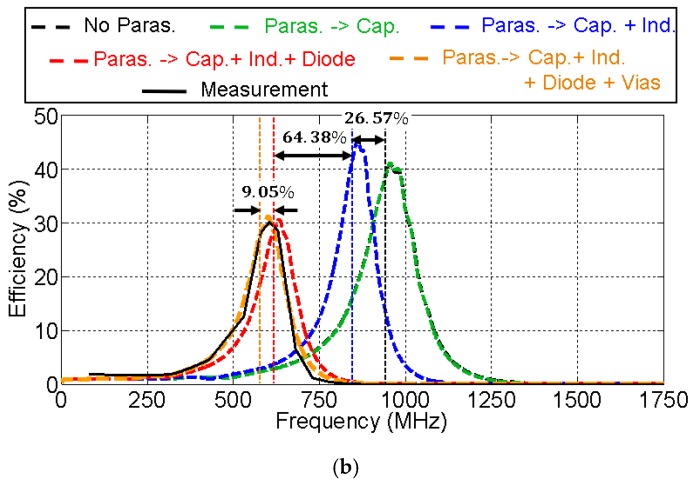
Model for the parasitic elements in the test half–wave rectifier (**a**) and their relevance in the efficiency of the circuit (**b**). The values of the components are: $L_{v}^{\prime }=1.20\;\mathrm{nH},$ $C_{L}^{\prime }=2.7\;\mathrm{pF},$ $L_{p1}^{\prime }=0.38\;\mathrm{nH},\;L_{L}^{\prime }=47\;\mathrm{nH},$ $C_{p}^{\prime }=0.14\;\mathrm{pF},$ $C_{pd}=0.75\;\mathrm{pF},\;C^{\prime }=27\;\mathrm{pF},\;L_{p2}^{\prime }=0.04\;\mathrm{nH},\;\mathrm{and}\;R_{L}^{\prime }=8\;k\Omega .$ The input power was $P_{in}=0$ dBm. Black and green dashed lines overlap.

Figure 10 and Figure 11 show the effect of the parasitic elements in the CW multiplier and the half–wave rectifier, respectively. The frequency displacement they cause (black dashed line versus black solid line) is noticeable in both circuits. However, note the good agreement between the complete simulation model (orange dashed line) and the measurement (black solid line). 

The contribution of each parasite to the total displacement has been quantified, and some conclusions were extracted. In both cases, the contribution of the parasitic inductance associated with the capacitors (green dashed line) was completely negligible. On the other hand, the parasitic shunt capacitance $C_{pd}$ associated with the diode package (red dashed line) was the most harmful element, with a total contribution over of 64% to the total frequency displacement in both circuits. In that sense, recent works have shown the benefits of two-dimensional materials, e.g., MoS_2_ (molybdenum disulfide) [23,24], being applied to RF electronics. In particular, [24] presents a MoS_2_-based Schottky diode used as a rectifier in a RF energy harvesting system. To explain the different behavior of traditional Schottky diodes though, the junction and parasitic capacitances of this MoS_2_-based diode are in the order of 20 fF, 35 times less than those shown in Figure 10 and Figure 11. Since the parasitics of the diode are the most damaging, the performance of the rectifiers could be potentially improved with the development of MoS_2_ diodes. Additionally, their cutoff frequency is also higher, which allows one to reduce losses at higher operating frequencies. As a comparison, the MoS_2_-based diode presented in [24] has a cutoff frequency of 10 GHz (zero external bias), while the HSMS 2822 diode utilized in this work only reaches up to 4 GHz.

The contribution of the parasitic capacitance associated with inductors in the *L* matching networks is represented in blue in Figure 10b and Figure 11b. In the case of the CW multiplier (Figure 10b), the frequency displacement they cause (9.67%) is lower compared to other parasitic terms, since the SRFs of the 4.3 nH and 8.2 nH inductors (see Table 1) are large. In addition, the contribution to the frequency displacement of the parasitic inductance associated with via holes (yellow curve in Figure 10b) is higher in this case, 22.25%. On the other hand, Figure 11b shows that the contribution, in the half-wave rectifier, of the parasitic capacitance associated with the inductor in the *L* matching network (blue curve), is appreciable—26.57%. However, the contribution of the parasitic inductance associated with the via holes (yellow curve) is less, 9.05%. 

As a comparison between both circuits, the inductor used in the *L* network of the half-wave rectifier possesses a lower SRF than the inductors of the CW multiplier (see Table 1). Thus, the parasitics of the latter are less harmful. Conversely, the contribution of the parasitic inductance associated with via holes is more prominent in the CW multiplier, despite its value being approximately a third of the inductance $L_{L1}$ and a sixth of $L_{L2}.$ However, since the parasitic capacitors $C_{p1}$ and $C_{p2}$ may be neglected in this circuit, there would be three parasitic inductances $L_{v}$ in series, and the sum of their values (3×1.3 nH) is of the order of $L_{L1}=4.3$ nH.

**Figure 12:**
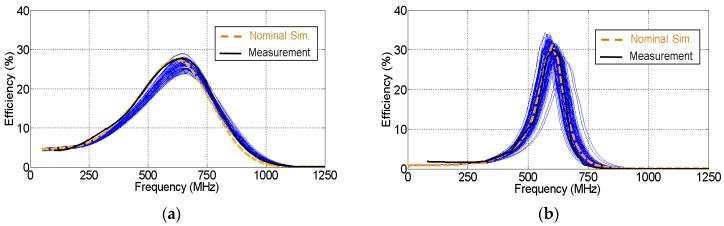
Monte Carlo analysis on the variability of the components in (**a**) the Cockcroft–Walton multiplier and (**b**) the half–wave rectifier. The nominal simulation and the measurements are also plotted.

Figure 12 presents a Monte Carlo analysis on the effect of the variability of the components on the circuit efficiency. The analysis was applied to the CW multiplier and half-wave rectifier of Figure 10 and Figure 11, respectively, after all the parasitics were included in the model. Capacitors ($C,\;C^{\prime },\;\mathrm{and}\;{C_{L}}^{\prime }$), inductors ($L_{L1},\;L_{L2},\;\mathrm{and}\;{L_{L}}^{\prime }$) and loads ($R_{L}\;\mathrm{and}\;{R_{L}}^{\prime }$) were assumed to follow a Gaussian distribution of standard deviation ±5% from their nominal values. Figure 12a presents the Monte Carlo analysis on the CW multiplier, and Figure 12b shows one on the half-wave rectifier. The minimum deviation observed in both figures between the nominal simulation and the measurement is within the variability range of the components. As observed, the frequency deviation caused by the variability of the components was much smaller than the one caused by the parasitics in Figure 10 and Figure 11. It can be noticed in Figure 12 that the half-wave rectifier is more sensitive to deviation in the components than the CW multiplier. This is due to variations in the capacitor ${C_{L}}^{\prime }$ in the *L* network of the half-wave rectifier. 

It is worth noticing that, in general terms, the most harmful parasitics are associated with lumped elements and not with the PCB. Even in the worst scenario, the CW multiplier, the effect of the vias was not so important and easily neutralized with the circuit model. A particularly important conclusion can be extracted: despite us using a perforated PCB (perfboard), considered a low-quality board in RF, its parasitics are not especially harmful at these frequencies. Therefore, if carefully chosen, low-quality RF PCBs can be utilized at frequencies below 1 GHz in order to ease the design and reduce costs of the rectifier stage in a harvesting system.

### 5.3. Final Circuits

**Figure 13:**
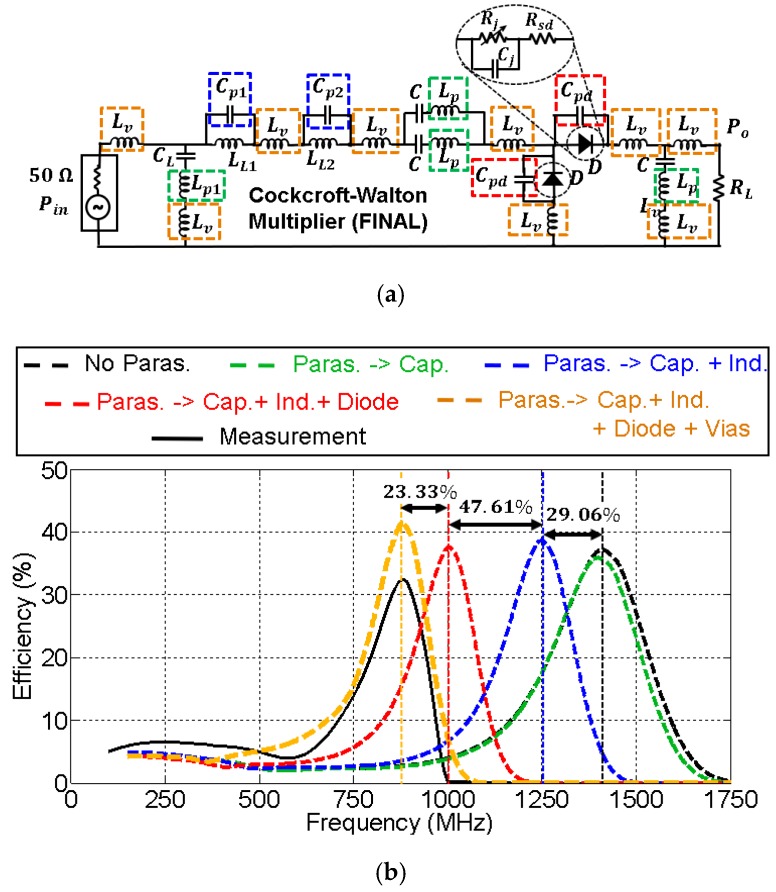
Model for the parasitic elements in the final Cockcroft–Walton multiplier (**a**) and their relevance in the efficiency of the circuit (**b**). The values of the components are: $L_{v}=1.30\;\mathrm{nH},\;C_{p}=4.7\;\mathrm{pF},$ $L_{p1}=0.078\;\mathrm{nH},$ $C_{p1}=0.10\;\mathrm{pF},\;L_{L1}=4.3\;\mathrm{nH},$ $C_{p2}=0.19\;\mathrm{pF},$ $L_{L2}=8.2\;\mathrm{nH},$ C=33 pF, $L_{p}=0.16\;\mathrm{nH},$ $C_{pd}=0.75\;\mathrm{pF},$ and $R_{L}=2.34\;k\Omega .$ The input power was $P_{in}=0$ dBm.

**Figure 14:**
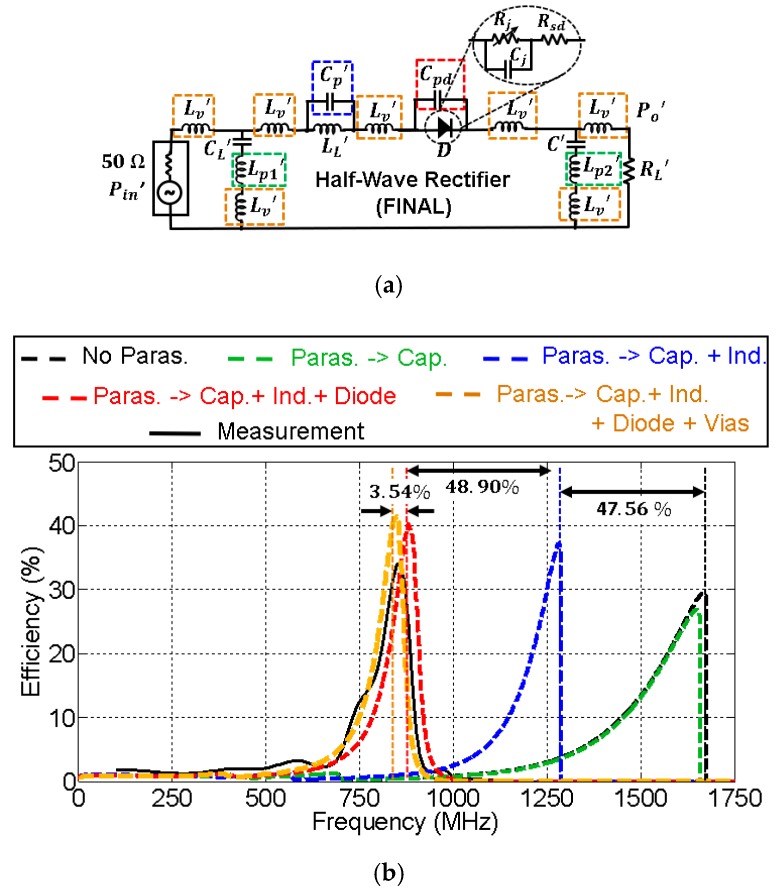
Model for the parasitic elements in the final half-wave rectifier (**a**) and their relevance in the efficiency of the circuit (**b**). The values of the components are: $L_{v}^{\prime }=1\;\mathrm{nH},$ $C_{L}^{\prime }=4.7\;\mathrm{pF},$ $L_{p1}^{\prime }=0.078\;\mathrm{nH},\;L_{L}^{\prime }=27\;\mathrm{nH},$ $C_{p}^{\prime }=0.21\;\mathrm{pF},$ $C_{pd}=0.75\;\mathrm{pF},$ $C^{\prime }=27\;\mathrm{pF},\;L_{p2}^{\prime }=0.04\;\mathrm{nH},\;\mathrm{and}\;R_{L}^{\prime }=8\;k\Omega .$ The input power was $P_{in}=0$ dBm.

With the use of the circuit model that contemplates the effect of the parasitics, the CW multiplier and the half-wave rectifier were redesigned to center their operation frequency at 870 MHz. Figure 13 and Figure 14 show the parasitic model of the final circuits. The frequency displacement caused by the parasitic elements is noticeable: more than 700 MHz from the ideal response. Again, the most damaging parasitic contribution comes from the shunt capacitance of the diode. The parasitic capacitance of the inductor had a bigger impact on the performance of the final circuits than for the test circuits since the operating frequency was higher (900 MHz versus 600 MHz). 

**Figure 15:**
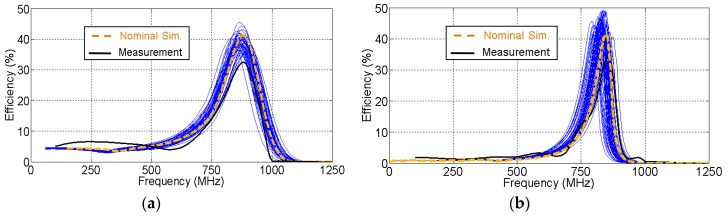
Monte Carlo analysis on the variability of the components in the final circuits: (**a**) Cockcroft–Walton multiplier and (**b**) half-wave rectifier. The nominal simulation and the measurements are also plotted.

A slight difference in amplitude existed between the simulated and measured efficiencies in Figure 13 and Figure 14. In order to study the cause of this difference, a Monte Carlo analysis was performed on the variability of the components. Capacitors, inductors and the load were assumed to be random variables that follow a Gaussian distribution of standard deviation 5% from their nominal values. As seen in Figure 15, the measurement curve fits within the selected variability range in simulation. As a result, the difference in efficiency could be explained by the effect of the component variability.

**Figure 16:**
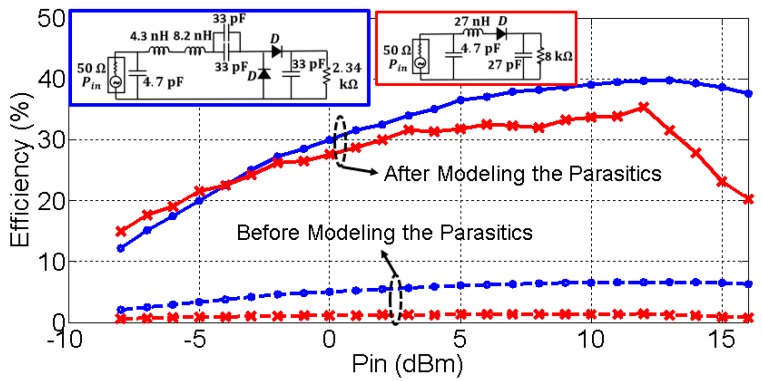
Efficiency (measured at 870 MHz) as a function of the input power in the CW multiplier (blue) and the half-wave rectifier (red).

Figure 16 represents the efficiencies of both circuits (see their schematic) as a function of the input power. As it can be seen, the efficiency rapidly drops below 5% due to the frequency displacement the parasitics cause. After including them in our circuit model, the rectifier efficiency rose to over 30% in both circuits, which is approximately twice the values (15%–20%) typically shown in the literature [10,12,13]. The nonlinearity of both circuits is appreciated in Figure 16. In a linear scheme, the efficiency curve would be completely flat. However, the efficiency response is flatter for the half–wave rectifier compared to the CW. This implies that the Cockcroft–Walton multiplier has a higher nonlinear dependence with respect to the input power, compared to the half–wave rectifier. Additionally, there was a drop in the efficiency of both circuits above 13 dBm. The diodes are saturated by high input powers and the performance of the rectifiers degrade. The drop is more pronounced in the case of the half–wave rectifier (red curve) due to the non-linearity effect in the circuit.

### 5.4. Performance Comparison of Passive Rectifiers

Finally, Table 2 summarizes a performance comparison among previous rectifier designs published in the literature [25,26,27,28,29,30,31]. For a fair comparison, only passive rectifiers that operate with an input power close to 0 dBm (1 mW) were considered. Note that active rectifiers are able to reach efficiency values of 75%–80%. However, they are not suitable for energy harvesting, and hence, were not considered in the study, since they need an external power supply.

Most of the passive implementations presented make use of half-wave and Cockcroft–Walton schemes, as they usually offer the least losses by using the minimum number of diodes. To the best of our knowledge, the highest efficiency found in the literature (from a passive rectifier) was recently presented in [29], reaching a value of 60%. Conversely, the vast majority of works present low rectifier efficiencies, close to 20% or even much lower in some cases. As a consequence, the detailed study on the parasitic effects presented in this work could help to significantly improve the performance of future rectifier circuits and RF energy harvesting systems.

## 6. Conclusions

This work presents guidelines for the design of efficient rectifier circuits in RF energy harvesting. Some advice was given in order to face the problems associated with the nonlinearity and parasitic elements of the circuits. Without loss of generality, two different configurations were tested: a CW multiplier and a half-wave rectifier. For a given load $Z_{L},$ it was shown that there is an optimal source impedance $Z_{s}$ that maximizes the rectifier efficiency, which was used to design an optimal matching circuit. Subsequently, the contribution of each parasitic element to the total frequency displacement was quantified and the most harmful parasitics were identified. In summary, the following conclusions have been extracted from the analyses:

1. The most harmful parasitics come from the components and not from the PCB. Therefore, if carefully chosen, cheaper PCBs can be utilized in order to reduce costs.
2. Although the diode was known to be the limiting component in terms of losses, this work has demonstrated that it also causes a large deviation with respect to the expected frequency response. Actually, it has the most harmful parasitic element, contributing two-thirds of the total frequency displacements in both Cockcroft–Walton and half–wave circuits. In that sense, future MoS_2_ diodes could potentially help to improve the efficiency of rectifier circuits, since their parasitics are shown to be very low [24] compared to traditional Schottky diodes.
3. The parasitic inductance associated with the capacitors is completely negligible. This fact allows one to use cheaper capacitors and reduce costs. It also allows one to use higher values of the capacitor in the rectifier stage, in order to reduce the DC output ripple when feeding the sensor. Note that the choice of this capacitor is a trade-off between the output ripple of the circuit, and its self-resonant frequency (SRF). The higher the value of the capacitor is, the lower the output ripple and the higher the parasitics. However, they do not affect the behavior of the circuit.

Finally, the circuit model of the parasitic elements was used to redesign both circuits, without considering the parasitics. The efficiency did not exceed the 5% in both circuits. After modeling them, the efficiency was shown to be over the 30%, twice the value compared to the passive rectifiers typically shown in the literature.

## Figures and Tables

**Table 1:** SRF and parasitics of the lumped elements.

Model	Value	SRF (GHz)	Parasitic
4,841,372 (Fair-Rite)	33 nH	1.50	0.34 pF
106–909 (Murata)	8.2 nH	4.00	0.19 pF
795–8290 (TDK)	4.3 nH	7.64	0.10 pF
464–6773 (AVX)	33 pF	2.20	0.16 nH
532–2945 (TE Connect.)	47 nH	1.96	0.14 pF
CW160,808 (Bourns)	27 nH	2.10	0.21 pF
2,310,325 (Multicomp)	2.7 pF	4.97	0.38 nH
ATC 500S (ATC)	4.7 pF	8.32	0.078nH
2,809,454 (Kemet)	27 pF	4.84	0.040 nH

**Table 2:** Performance comparison of passive rectifiers.

Ref.	Freq. (GHz)	Rectifier Circuit	Input Power (dBm)	Circuit Efficiency (%)
[10]	−	Half–Wave	4	20
[25]	2.4	CW	0	21
[26]	0.95	CMOS	0	9
[27]	−	CW	0	21
[28]	0.87	CW	0	30
[29]	0.85	CW	0	60
[30]	2.4	Half–Wave	0	14
[31]	2.4 5.5	Full–Wave	0	36 5
**This Work**	0.87	CW Half-Wave	0	30 27.5
